# Best Practice Recommendations for the Assessment, Prevention and Treatment of Vitamin D Deficiency in Türkiye: A 2026 Update in a Setting with Limited Mandatory Food Fortification

**DOI:** 10.3390/nu18111665

**Published:** 2026-05-22

**Authors:** Dilek Gogas Yavuz, Ömercan Topaloğlu, Mutlu Güneş, Alper Gürlek, Ayşe Kubat Üzüm, Zafer Pekkolay, Zeynep Cantürk, Zeliha Hekimsoy, Özen Öz Gül, Refik Tanakol

**Affiliations:** 1 Department of Endocrinology and Metabolism, Marmara University School of Medicine, İstanbul 34710, Türkiye; 2 Department of Endocrinology and Metabolism, Zonguldak Bülent Ecevit University Medical Faculty, Zonguldak 67100, Türkiye; omercan.topaloglu@beun.edu.tr; 3 Department of Endocrinology and Metabolism, Health Sciences University Bursa Yüksek İhtisas Training and Research Hospital, Bursa 16310, Türkiye; drmutlugunes@gmail.com; 4 Department of Endocrinology and Metabolism, Hacettepe University Faculty of Medicine, Ankara 06100, Türkiye; agurlek2001@yahoo.com; 5 Department of Endocrinology and Metabolism, İstanbul School of Medicine, İstanbul University, İstanbul 34093, Türkiye; ayse.kubat@istanbul.edu.tr; 6 Department of Endocrinology and Metabolism, Dicle University School of Medicine, Diyarbakır 21280, Türkiye; zafer.pekkolay@dicle.edu.tr; 7 Department of Endocrinology and Metabolism, Kocaeli University Faculty of Medicine, Kocaeli 41380, Türkiye; zeynepcanturk@hotmail.com; 8 Department of Endocrinology and Metabolism, Manisa Celal Bayar University Faculty of Medicine, Manisa 45010, Türkiye; zhekimsoy@hotmail.com; 9 Department of Endocrinology and Metabolism, Bursa Uludağ University Medical School, Bursa 16059, Türkiye; ozenoz@uludag.edu.tr; 10 American Hospitals in İstanbul, İstanbul, 34365, Türkiye; rtanakol@gmail.com

**Keywords:** vitamin D deficiency, 25-hydroxyvitamin D, cholecalciferol, vitamin D supplementation, Türkiye

## Abstract

Background: Vitamin D deficiency is a common global health problem and remains highly prevalent in Türkiye, where limited food fortification and heterogeneous clinical practices contribute to variability in testing and supplementation strategies. Aims: To provide Türkiye-specific best practice recommendations for defining clinically relevant serum 25-hydroxyvitamin D [25(OH)D] thresholds, identifying adult risk groups for targeted testing, and recommending evidence-based prevention, treatment, and monitoring approaches while minimizing under-treatment and inappropriate high-dose use. Methods: This national expert consensus document was developed by endocrinologists from across Türkiye using a structured, modified Delphi methodology. Draft statements informed by systematic literature reviews were rated via online surveys using a 9-point Likert scale, followed by two Delphi rounds and a face-to-face consensus meeting in İstanbul in October 2025. Results: Recommendations addressed sun exposure, laboratory assessment, screening, supplementation, treatment, and follow-up. Serum 25(OH)D <20 ng/mL was defined as deficiency and <12 ng/mL as severe deficiency, with a target range of 20–50 ng/mL. Routine population-wide screening was not recommended; instead, targeted testing in high-risk adults and symptom-driven biochemical evaluation were endorsed. Empiric supplementation was recommended for selected high-risk groups, with cholecalciferol as the preferred agent. Higher individualized doses were suggested in obesity or malabsorption, while loading regimens were reserved for specific clinical indications, such as severe deficiency or certain medical conditions that impair vitamin D metabolism. Reassessment of 25(OH)D at 8–12 weeks was recommended. Conclusion: These consensus-based recommendations provide a practical, context-specific framework for assessing, preventing, treating, and monitoring vitamin D deficiency in adults in Türkiye.

## 1. Introduction

Vitamin D is a secosteroid hormone that plays a central role in calcium–phosphate homeostasis and bone mineralization and also exerts pleiotropic effects on the immune, cardiovascular, metabolic, and reproductive systems via widespread expression of the vitamin D receptor (VDR) [1,2]. Because only a limited number of foods naturally contain or are fortified with vitamin D, most of the body’s requirement is met by endogenous synthesis in the skin under ultraviolet B (UVB) radiation [3,4].

Consequently, vitamin D status is determined by the complex interaction of latitude, season, clothing, sun-exposure behavior, skin pigmentation, age, diet, and supplement use [3,5].

Over the past two decades, vitamin D deficiency has been recognized as a major global public health problem across all age groups, including populations living at low latitudes where sunlight is abundant [3,6,7]. It is estimated that up to one billion people worldwide are deficient or insufficient, with serum 25-hydroxyvitamin D [25(OH)D] concentrations frequently below thresholds considered adequate for musculoskeletal health [1,3,6]. Observational and mechanistic data link low 25(OH)D concentrations not only to classical skeletal outcomes—rickets, osteomalacia, osteoporosis, and fragility fractures—but also to increased risks of falls, infections, cardiometabolic disorders, and other chronic non-communicable diseases [1,2,3,5,6,7,8,9,10].

Despite broad consensus on the need to prevent severe deficiency, controversies remain regarding optimal 25(OH)D thresholds and treatment strategies. Authorities vary between recommending serum 25(OH)D concentrations <20 ng/mL for the general population, as reflected in IOM and Endocrine Society guidance, and <30 ng/mL for older adults and high-risk groups, particularly in the context of osteoporosis and fracture prevention [8,9,10].

Vitamin D Deficiency in Türkiye is a High-Prevalence Public Health Issue. Unlike in many Northern European and North American countries, Türkiye has not implemented systematic vitamin D fortification policies [3,4]. Türkiye has very limited mandatory food fortification, and habitual dietary vitamin D intake is generally low [11]. At the same time, awareness of vitamin D has increased, leading to widespread prescription and over-the-counter use of vitamin D preparations, particularly in outpatient and hospital-based settings. In the absence of nationally harmonized, context-specific guidance, the situation has resulted in considerable heterogeneity in testing strategies, treatment thresholds, and dosing regimens, ranging from under-treatment in high-risk individuals to inappropriate or excessive supplementation in those without clear indications [12,13]. Differences among international guidelines, which cater to populations with varying baseline intakes, fortification policies, and healthcare structures, further amplify this variability [8,9,10].

These “Best Practice Recommendations for the Assessment and Management of Vitamin D Deficiency in Türkiye” have been developed to address this gap. The objectives are to define clinically relevant thresholds for vitamin D deficiency and insufficiency; identify high-risk groups and indications for testing; recommend evidence-based regimens for prevention and treatment of deficiency in adults, including special populations; and minimize both under-treatment and inappropriate high-dose use of vitamin D in Türkiye. The overarching aim is to provide a pragmatic, context-specific framework for optimizing vitamin D status in adults and improving musculoskeletal and overall health outcomes in the Turkish population.

## 2. Materials and Methods

### 2.1. Expert Panel Composition

The expert panel included 10 endocrinologists with expertise in vitamin D and metabolic bone disease from tertiary centers across Türkiye. All panelists participated in each Delphi round and the final face-to-face meeting, and all are authors of this manuscript.

### 2.2. Delphi Methodology

A modified Delphi method was used to develop and refine the statements in this document. The process comprised three phases: preparation, two rounds of online voting, and a final face-to-face consensus meeting. The Delphi approach uses anonymous, iterative ratings, which are repeated assessments made without revealing the identities of the participants, along with controlled feedback and aggregation of responses to minimize bias and reduce the influence of individual panelists.

For each round, panel members rated every statement using an electronic survey platform SurveyMonkey^®^ (Momentive Inc., San Mateo, CA, USA) on a 9-point Likert scale (1 = strongly disagree to 9 = strongly agree), consistent with Delphi and RAND/UCLA methodologies. Consensus was predefined as ≥75% of ratings in the 7–9 range (“agree/strongly agree”). It was defined as ≥75% = consensus achieved threshold, and 100% consensus = unanimous agreement among panelists. Therefore, occasional low or neutral ratings did not preclude achievement of consensus when the predefined threshold of ≥75% agreement was met. Individual dissenting opinions were not incorporated as separate recommendations but were transparently reflected in the supplementary scoring tables. Statements below this threshold were revised and revoted on [14,15].

After defining the aims and scope, a 10-member expert panel conducted systematic literature reviews and drafted statements based on international and national guidance, focusing on practical aspects of vitamin D assessment, prevention, and treatment in Türkiye. The first online round (February 2025) informed revisions for the second round, in which panelists received anonymized group feedback and their prior scores before re-rating. Statements meeting the consensus criterion were accepted; remaining items were discussed at an in-person meeting in İstanbul on 10 October 2025, attended by all panelists. Following structured discussion and wording refinement to ensure applicability to Turkish practice, revised statements were re-voted using the same criteria, achieving consensus for all final statements. Statements, overall agreement, the voting scale, level of agreement, and number of votes are provided in Supplementary Table S1.

## 3. Results and Discussion

These guidelines are formulated from an endocrine-specialist perspective. Although a limited number of panelists assigned low agreement scores (2–3) to certain statements, all accepted recommendations fulfilled the predefined Delphi consensus criterion (≥75%), supporting their inclusion as consensus-based recommendations. Nevertheless, we acknowledge that these individual disagreements may reflect ongoing expert-level variability and contextual differences in clinical interpretation.

### 3.1. Sun Exposure and Cutaneous Vitamin D Synthesis in Türkiye

Cutaneous vitamin D_3_ is generated when 7-dehydrocholesterol in the epidermis absorbs ultraviolet-B (UVB) photons in the 290–315 nm range and, after thermal photoisomerization at typical warm-season skin temperatures (≥25 °C), is converted to previtamin D_3_ and subsequently cholecalciferol [16,17].

In Türkiye (36–42° N), modeling of the UV index and vitamin-D–weighted UVB for İstanbul and Antalya shows that solar elevation is sufficient for effective cutaneous vitamin D synthesis from early spring to early autumn, with an extended “vitamin D season” roughly between April and September–November depending on latitude and atmospheric conditions [18]. Clinical and biophysical data indicate that approximately 0.5 minimal erythemal dose (MED) of UVB delivered to a limited body surface (e.g., arms and legs) is adequate to trigger substantial vitamin D synthesis, but the exact dose required is strongly influenced by time of day, season, latitude, skin pigmentation, age-related decline in cutaneous 7-dehydrocholesterol, clothing habits, and air pollution [17,19,20].

Observational data from Niğde and other Turkish provinces show peak 25(OH)D concentrations in late spring and summer despite high national prevalence of deficiency [21,22]. However, because erythema and photocarcinogenesis share a similar UVB action spectrum, prolonged sun exposure without sunscreen beyond about 15–30 min near solar noon, especially in fair-skinned individuals, substantially increases the cumulative risk of actinic damage and skin malignancies, and thus vitamin D–oriented photoprotection strategies should aim for brief, suberythemal, nonsunscreened exposures followed by appropriate sun protection [1,23,24]. We suggest that at the latitude of Türkiye, a recent UV-index–based model estimated that for 25% body surface exposure (roughly arms and legs) during the active UVB period between April and September, sub-erythemal sun exposure between about 10:00 and 16:00 for roughly 10–30 min—consistent with empirical estimates of 12–30 min 3–4 times per week across different Fitzpatrick skin types—can provide a daily equivalent of ~1000 IU vitamin D_3_ and is therefore sufficient to maintain physiological requirements in otherwise healthy individuals [18,20]. Our statements for vitamin D synthesis for Türkiye are shown in Table 1.

### 3.2. Evaluation and Interpretation of Serum 25(OH)D Concentrations

25(OH)D is the established biomarker for assessing vitamin D status. It reflects both cutaneous synthesis and dietary intake and is widely accepted as the most reliable indicator in both clinical and research settings [10].

The preferred method for 25(OH)D quantification is liquid chromatography–tandem mass spectrometry (LC-MS/MS) due to its superior accuracy and reproducibility. In the absence of mass spectrometry, standardized immunoassays such as chemiluminescent or immunoenzymatic methods may be used, provided they are validated and aligned with international standards, such as those of the Vitamin D Standardization Program (VDSP) or IOF-endorsed protocols [17].

*Thresholds for Vitamin D Deficiency*: A serum 25(OH)D concentration <20 ng/mL is considered deficient and is associated with increased risk of skeletal disorders [10,25]. A concentration <12 ng/mL is defined as a severe deficiency, warranting prompt correction.

Concentrations ≥20 ng/mL are generally regarded as sufficient for maintaining bone and muscle health [10,25].

Concentrations between 30 and 50 ng/mL are suggested to be sufficient for extra-skeletal benefits, including immune regulation and cardiometabolic functions [25,26].

*Upper Limits and Potential Risk Zones*: Serum concentrations between 50 and 60 ng/mL are considered safe, although they exceed current targets and do not provide additional benefits in most populations [10].

Concentrations between 60 and 99 ng/mL fall into a gray zone, where the risk of adverse effects (e.g., hypercalcemia, hypercalciuria) may increase with rising concentrations [10]. A 25(OH)D concentration ≥100 ng/mL is classified as hypervitaminosis D, and may be associated with potential toxicity [27]. Concentrations ≥150 ng/mL define vitamin D intoxication, often associated with clinically significant hypercalcemia, suppressed PTH, and risk of renal and cardiovascular complications [27]. Vitamin D intoxication (toxicity) generally refers to markedly elevated serum 25(OH)D concentrations accompanied by hypercalcemia and related clinical manifestations. Although overt vitamin D intoxication is uncommon below 150 ng/mL, some individuals may develop hypercalciuria, nephrolithiasis, or other adverse effects at lower serum 25(OH)D concentrations, particularly with chronic high-dose supplementation. Our recommendations on the assessment of vitamin D concentrations are shown in Table 2.

### 3.3. Screening Strategy and Targeted Biochemical Evaluation for Vitamin D Deficiency in Adults in Türkiye

Vitamin D deficiency is highly prevalent among adults in Türkiye, with meta-analyses and nationwide hospital data indicating deficiency (25(OH)D < 20 ng/mL) in roughly 60% of the adult population [11,12].

Despite the high background prevalence, current evidence from randomized trials does not demonstrate a clear net benefit of population-wide screening for vitamin D deficiency, and major guidelines and epidemiologic studies; therefore, we do not recommend routine 25(OH)D testing in asymptomatic, low-risk adults [10,28,29].

In accordance with these international recommendations, we do not advise routine community-based screening for vitamin D deficiency in the general adult population in Türkiye [10,28,30].

Instead, measurement of serum 25(OH)D should be considered in individuals or patient groups at increased risk of deficiency and expected to derive particular benefit from vitamin D treatment, as listed in Table 3 and endorsed by the whole consensus group [10,17,30].

Serum 25(OH)D assessment is appropriate in adults presenting with symptoms compatible with vitamin D deficiency, including unexplained musculoskeletal or bone pain, proximal muscle weakness, muscle cramps, gait or balance problems, and atraumatic (fragility) fractures [31].

In adults with serum 25(OH)D concentrations <12 ng/mL and in patients with 25(OH)D concentrations between 12 and 20 ng/mL who have clinical features potentially attributable to vitamin D deficiency, we recommend measuring serum total calcium, phosphate, intact parathyroid hormone (iPTH), and alkaline phosphatase to evaluate for secondary hyperparathyroidism and osteomalacia [32].

Although the daily dietary vitamin D intake in Türkiye is unknown, it is recommended to take 600 IU as a supplement because adult participants’ diets are not fortified with vitamin D. Given the age-related decline in cutaneous vitamin D synthesis and intestinal absorption, together with the high prevalence of deficiency in older adults in Türkiye, we recommend ensuring a total vitamin D intake (from diet and supplements combined) of 800 IU/day in individuals aged ≥65 years, consistent with RDA-based international recommendations for this age group [33,34]. Statements on screening strategy and targeted biochemical evaluation for vitamin D deficiency in adults in Türkiye are shown in Table 4.

### 3.4. Empirical Vitamin D Supplementation in High-Risk Adult Groups

In older adults, pregnant women, individuals with prediabetes, and persons at increased risk of vitamin D deficiency, we recommend empiric vitamin D supplementation to prevent deficiency and to maintain vitamin D concentrations within the target range. *Empiric vitamin D supplementation* refers to oral vitamin D intake that exceeds the RDIs and is implemented without prior testing for serum 25-hydroxyvitamin D. Vitamin D doses used in the available clinical trials varied considerably; therefore, the optimal dosing regimen remains uncertain [10]. Statements shown in Table 5. In the Turkish setting, where vitamin D–fortified foods are scarce and background deficiency is common, empirical vitamin D supplementation (supplementation that exceeds the DRI and is initiated without prior 25(OH)D testing) is recommended for selected high-risk groups. Empiric supplementation with 2000 IU/day should primarily be considered for selected high-risk groups, particularly in settings where routine screening or food fortification is limited. Adherence, potential overtreatment, and cost-effectiveness should be considered in clinical decision-making, and further population-specific cost-effectiveness studies from Türkiye are needed to better support universal implementation.

**Individuals at risk of vitamin D deficiency include adults who are at an increased risk, as mentioned in **Table 3**.** If it is not possible to measure 25OHD concentrations, empirical supplementation with 800–2000 IU/day of vitamin D is recommended to maintain 25(OH)D at target concentrations.

**Older adults (≥75 years):** Because of the high prevalence of vitamin D deficiency in elderly populations and the age-related decline in cutaneous synthesis and intestinal absorption, we recommend empirical vitamin D supplementation in all individuals aged 75 years and older [34].

In the general population aged 75 years and over, an additional 2000 IU/day of vitamin D can be given empirically on top of the 800 IU/day that should ideally be obtained from food, to maintain serum 25(OH)D within the target range (≥20–30 ng/mL, depending on the guideline) and potentially reduce the risk of mortality and other adverse outcomes [10,35,36].

**Pregnancy and lactation:** Although routine population-based vitamin D screening during pregnancy is not recommended, targeted screening may be considered in pregnant women at high risk for vitamin D deficiency [10].

Given the association between low maternal vitamin D status and an increased risk of adverse pregnancy outcomes, including preeclampsia, intrauterine or neonatal death, preterm birth, and small-for-gestational-age birth, empirical vitamin D supplementation is recommended during pregnancy to improve maternal vitamin D status and potentially lower these risks [37].

In pregnant women, empirical vitamin D supplementation of 2000–2500 IU/day in addition to dietary intake is considered safe and effective for achieving adequate 25(OH)D concentrations, based on randomized trials using doses of 2000–4000 IU/day and expert-opinion–based recommendations that 1000–2000 IU/day is safe in pregnancy [38].

Because most clinical trials in pregnancy have used daily vitamin D regimens rather than intermittent large doses, pregnant women should preferably receive their vitamin D as a daily dose [37]. During lactation, a daily maternal vitamin D intake of about 2000 IU should be ensured to prevent deficiency in the mother and to support adequate vitamin D content in breast milk.

**Prediabetes:** In individuals with prediabetes, routine vitamin D screening with 25(OH)D measurement is not recommended in the absence of other established indications, consistent with recent Endocrine Society guidance and consensus statements that advise against population-based testing and instead favor a risk-stratified approach [10,30].

However, vitamin D supplementation in addition to lifestyle modifications (diet, weight loss, and physical activity) is recommended for adults with prediabetes to help prevent progression to type 2 diabetes and increase the probability of reversion to normoglycemia, as supported by randomized trials and meta-analyses showing reduced diabetes incidence and improved intermediate metabolic endpoints with vitamin D [39].

In the Endocrine Society 2024 guideline, vitamin D doses used in randomized trials involving adults with prediabetes ranged from 842 to 7543 IU/day in daily equivalents, with an estimated weighted average of approximately 3500 IU/day. Therefore, this value should be interpreted as a trial-derived average exposure rather than a universal recommended treatment dose. This dose can therefore be considered a reasonable empiric target for supplementation in adults with prediabetes who carry multiple risk factors for developing overt diabetes. The total intake of vitamin D should remain within accepted upper safety limits, and calcium metabolism must be monitored in high-risk individuals [10,30].

### 3.5. Treatment of Vitamin D Deficiency

A serum [25(OH)D] concentration less than 20 ng/mL should be treated to correct deficiency and prevent skeletal complications [1,2]. The therapeutic goal is to maintain serum 25(OH)D between 20 and 50 ng/mL, a range considered sufficient for almost all of the general population while avoiding potential risks associated with higher circulating concentrations [10,17,33].

In adults, cholecalciferol (vitamin D_3_) is recommended as the preferred preparation for both supplementation and treatment, because it is more effective than ergocalciferol (vitamin D_2_) in raising and sustaining serum 25(OH)D concentrations [40,41]. For most adults, a daily intake of 800–2000 IU/day of vitamin D is generally adequate to achieve and maintain sufficient 25(OH)D concentrations; this dose range is recommended both for routine supplementation and for the treatment of deficiency in the general adult population, with higher doses reserved for selected high-risk groups [40,41].

Vitamin D deficiency treatment should be initiated at a dose of 1500–2000 IU/day for individuals not receiving a loading dose, with continued supplementation recommended for lifelong if the underlying causes of deficiency are irreversible. Recent findings support this foundational treatment approach, demonstrating its efficacy and safety in both general and at-risk populations [10,17]. If the underlying contributors to deficiency cannot be adequately modified, long-term maintenance supplementation may be required to prevent recurrence.

In selected high-risk settings—including obesity, chronic use of medications that increase vitamin D catabolism (e.g., systemic glucocorticoids and enzyme-inducing antiepileptic drugs), and malabsorption syndromes—higher maintenance doses, often in the range of approximately 3000–6000 IU/day, may be necessary to attain comparable serum 25(OH)D concentrations; dosing should therefore be individualized [17].

In patients with persistent malabsorption, substantially higher dosing strategies have been reported, including higher daily dosing or intermittent high-dose regimens; however, such approaches should be reserved for well-defined clinical scenarios, implemented under specialist supervision, and accompanied by careful biochemical monitoring [42,43]. In this subgroup, if the response to cholecalciferol remains inadequate, hydroxylated vitamin D preparations (e.g., calcifediol) may be considered because of more predictable absorption and pharmacokinetics in some malabsorptive states, particularly in patients with conditions such as celiac disease or chronic pancreatitis that impair nutrient absorption [42,43]. For routine daily supplementation in adults, many authorities cite a tolerable upper intake level of 4000 IU/day; doses exceeding this threshold should generally be used cautiously, for specific indications, and with appropriate surveillance for potential adverse effects [44,45].

Overall, the therapeutic aim is to maintain serum 25(OH)D within a reasonable target range—commonly around 20–50 ng/mL depending on the guideline and clinical context—while avoiding sustained higher concentrations that may increase the likelihood of harm [17]. Treatment, rapid correction modalities, and follow-up consensus statements are shown in Table 6.

A vitamin D loading regimen is not routinely required in all adults with deficiency; instead, short-term use of higher doses should be reserved for situations in which rapid clinical correction of vitamin D deficiency is necessary [40,41]. Rapid correction is also indicated in individuals with osteomalacia and very low 25(OH)D concentrations (<12 ng/mL); in patients with osteoporosis at very high fracture risk; and in those presenting with secondary hyperparathyroidism and/or hypocalcemia, in whom prompt restoration of vitamin D status is needed to stabilize calcium–phosphate homeostasis and to reduce skeletal risk [41]. After a loading dose, a maintenance dose of 800–2000 IU/day is suggested lifelong if the situation of vitamin D deficiency is unresolved (Table 6).

Loading regimens are not required in all adults with vitamin D deficiency; however, they may be considered when a more rapid biochemical correction is clinically desirable, particularly in the setting of marked deficiency (e.g., very low 25(OH)D), suspected secondary hyperparathyroidism, or hypocalcemia, where timely restoration of vitamin D status may be relevant to clinical management [46]. The loading protocol of cholecalciferol, 50,000 IU once weekly for 8 weeks or an equivalent, was found to be safe and effective in non-obese subjects [47]. Alternative regimen may consist of 6000–10,000 IU daily, followed by maintenance therapy of 800–2000 IU/day [42].

Serum 25(OH)D concentrations in obese adults are lower than in those with normal body weight due to volumetric dilution and decreased liver 25-hydroxylase activity. Obese patients may require higher doses of vitamin D, 3000–6000 IU/day, to reach the target 25(OH)D concentration compared to patients with normal body weight [48].

Based on the serum results, clinicians should decide whether to continue with the current treatment regimen or transition the patient to a maintenance dose. Recent clinical reviews have highlighted the importance of individualized treatment adaptation, particularly for patients with complex needs or long-term deficiency [47,48]. Different dosing schedules, such as daily, biweekly (every 15 days), or monthly, have been shown to be equally effective and safe. This lets doctors customize treatment to fit each patient’s needs or likelihood of sticking with it. This flexibility is valuable, especially in populations where daily compliance is challenging, such as those with busy lifestyles, cognitive impairments, or socioeconomic barriers that make it difficult to adhere to a daily medication schedule [46].

The amount of vitamin D needed to effectively treat vitamin D deficiency depends partly on the baseline concentration of serum 25(OH)D, as well as the individual’s capacity to absorb vitamin D, their liver’s capacity to convert vitamin D to 25(OH)D, and, to some extent, unknown genetic determinants. Therefore, induction and maintenance therapy should be individualized, taking into account baseline vitamin D concentrations, body weight, and absorption capacity [17,48].

In Türkiye, oral vitamin D preparations are commercially available in capsule, drop, and tablet formulations. Capsule strengths include 1000 IU, 2000 IU, 5000 IU, 10,000 IU, 20,000 IU, and 50,000 IU. Drop formulations are available as 50,000 IU/15 mL, 50,000 IU/10 mL, and 150,000 IU/10 mL. Tablet formulations are available as 2000 IU and 20,000 IU. In addition, an intramuscular preparation is available as a 300,000 IU/1 mL ampoule.

Clinical studies show that capsules, drops, and tablets offer similar efficacy when used at equivalent doses [48,49]. From a physiological standpoint, daily cholecalciferol supplementation most closely mimics continuous endogenous vitamin D input and has been associated with higher systemic exposure to 25(OH)D than intermittent dosing in some studies [17].

Parenteral administration should be reserved for patients who have severe gastrointestinal malabsorption, resistance to oral therapy, or are critically ill—scenarios where oral formulations are not viable or effective [48].

However, alternative bolus dosing regimens—such as seasonal or yearly high doses of 300,000 to 600,000 IU—offer a more convenient supplementation option, though they raise significant safety concerns, particularly the increased risk of falls and fractures, especially among the elderly, and are not recommended [49,50].

Implementing an individualized treatment plan that accounts for the patient’s preferred frequency of dosing—whether daily, weekly, or monthly—may significantly improve adherence to vitamin D therapy. Adherence is a crucial factor in long-term outcomes, especially in chronic deficiency management, and aligning treatment with patient lifestyle and habits increases success rates.

### 3.6. Monitoring and Follow-Up

Serum 25(OH)D should be remeasured 8–12 weeks after the loading dose or initiating therapy to assess biochemical response, as steady state is typically achieved within this interval. According to follow-up 25(OH)D results, the regimen should be continued, dose-adjusted, or transitioned to maintenance therapy, with subsequent monitoring tailored to baseline status and ongoing risk factors. (Table 6).

If serum 25(OH)D is within the target range (e.g., 20–50 ng/mL), transition to the maintenance dose or the current maintenance dose may be continued [30,48].

If serum 25(OH)D is 50–60 ng/mL, concentrations are generally not consistent with toxicity but exceed commonly recommended targets; total vitamin D intake (supplements, fortified foods, combined preparations) and dosing should be reviewed [33].

If serum 25(OH)D is 60–99 ng/mL (“gray zone”), concentrations are above ranges where benefit is consistently demonstrated and approach the Endocrine Society’s commonly cited upper limit (~100 ng/mL); supplementation should be temporarily withheld for 2–3 months with repeat testing after ~8–12 weeks, considering the kinetics and tissue storage of vitamin D metabolites [17,32,40]. Particular caution may be warranted in patients at risk for hypercalciuria or nephrolithiasis.

If serum 25(OH)D is ≥100 ng/mL, it is hypervitaminosis D; if serum 25(OH)D is ≥150 ng/mL, vitamin D intoxication (toxicity) should be considered, vitamin D should be discontinued, and clinical/biochemical evaluation (including serum calcium and renal indices) should be undertaken and management instituted as clinically indicated [51]. However, prolonged high-dose supplementation may pose risks even in the absence of overt hypercalcemia.

## 4. Strengths and Limitations

This consensus has several strengths. It was developed using a structured three-phase modified Delphi process with predefined criteria, combining anonymous iterative scoring, controlled feedback, and a final face-to-face meeting to resolve remaining disagreements. Full participation was achieved across all Delphi rounds. Statements were informed by international guidance and evidence summaries and were adapted to the Turkish context and routine clinical practice needs. Key limitations include the relatively small panel size (n = 10) and the lack of a multidisciplinary composition, which may reduce representativeness across diverse settings and regions. One limitation of this consensus statement is that, although the predefined Delphi consensus threshold was achieved, one panel member expressed disagreement regarding the statement on vitamin D synthesis and sun exposure in Türkiye.

## 5. Conclusions

Over the past two decades, vitamin D deficiency has emerged as a major global public health problem across all age groups. Türkiye represents a distinctive setting for vitamin D guidance because mandatory food fortification is minimal and habitual dietary intake is generally low, while public and professional awareness has driven widespread prescription and over-the-counter supplementation. In the absence of nationally harmonized, context-specific guidance, clinical practice has become highly heterogeneous—ranging from under-recognition and under-treatment in high-risk individuals to inappropriate testing and excessive dosing in those without clear indications. These gaps may be further widened when international guidelines are applied without adaptation to Turkish realities, leading to potential mismanagement of patient care and exacerbating health disparities within the population.

Accordingly, these best-practice recommendations were developed to provide a pragmatic, locally relevant framework for adult care in Türkiye. The objectives are to define clinically meaningful 25(OH)D thresholds; identify high-risk groups and clarify indications for targeted testing rather than population-wide screening; and recommend evidence-based prevention, treatment, and monitoring strategies, including practical dosing and formulation options, to minimize both under-treatment and inappropriate high-dose use while safeguarding against toxicity. The overarching aim is to optimize vitamin D status and musculoskeletal outcomes within real-world clinical practice.

## Figures and Tables

**Table 1:** Consensus Statements on Sun Exposure and Cutaneous Vitamin D Synthesis in Türkiye (36–42°N Latitude).

In Türkiye’s geographical region (36–42° north latitude), vitamin D synthesis takes place from early April to late September annually.
*Approximately 0.5 minimal erythemal doses of ultraviolet B radiation can be attained with direct sunlight exposure to the arms and legs; however, this is dependent upon factors such as time of day, season, latitude, skin type, advanced age, air pollution, and individual skin sensitivity*.
*At the latitude of Türkiye, during the active UVB period between April and September, exposing the skin without sunscreen between 10:00 and 16:00 for 12 to 30 min, 3–4 times per week (depending on skin type), is sufficient to meet physiological vitamin D requirements.*
*Sun exposure without sunscreen for longer than 15–30 min, depending on skin type, increases the risk of skin malignancies. Photoprotection strategies should aim for brief, sub-erythemal, nonsunscreened exposures followed by appropriate sun protection. To our knowledge, there is no large-scale nationally representative study defining the exact distribution of Fitzpatrick skin phototypes in the Turkish population. We generally recommend that the sun exposure should be limited under 30 min.*

Overall agreement, 100% consensus, is endorsed. Supplementary Table S1 displays the voting scale for consensus and the number of votes.

**Table 2:** Consensus Statements on the Assessment and Clinical Interpretation of Serum 25-Hydroxyvitamin D [25(OH)D] Concentrations in Adults.

Serum 25-hydroxyvitamin D [25(OH)D] is the accepted biomarker for evaluating vitamin D status. A 25(OH)D concentration of ≥20 ng/mL is considered sufficient to maintain musculoskeletal health. A 25(OH)D concentration of <20 ng/mL should be considered vitamin D deficiency. <12 ng/mL is classified as severe deficiency. 30–50 ng/mL is considered sufficient for extra-skeletal effects. 50–60 ng/mL are generally safe but exceed the recommended targets. 60–99 ng/mL fall into a gray zone, in which the risk of complications may increase with higher concentrations. ≥100 ng/mL is defined as hypervitaminosis D, and may be associated with risk of toxicity. ≥150 ng/mL is consistent with vitamin D intoxication (toxicity).

Overall agreement: 100% (consensus endorsed). Supplementary Table S1 provides the voting scale, level of agreement, and number of votes.

**Table 3:** Adult groups at increased risk of vitamin D deficiency.

*Screening for vitamin D deficiency should be considered in individuals with the following patient characteristics or clinical conditions:* **Older age:** adults aged **≥65 years** **High fall/fracture risk:** older adults with a history of **falls** or **low-trauma (fragility) fractures.** **Insufficient sunlight exposure:** **Homebound** individuals (e.g., disability-related confinement, immobility) Residents of **nursing homes/long-term care facilities** Individuals working **predominantly indoors** for prolonged hours (including **night-shift** workers in offices, hospitals, or factories) **Chronic debilitating conditions** associated with reduced mobility or limited outdoor activity **Obesity: BMI ≥ 30 kg/m^2^** **Chronic use of medications that alter vitamin D metabolism** (e.g., **antiepileptic drugs**, **systemic glucocorticoids**, **azole antifungals**, **antiretroviral therapy**) **Malabsorption states** (e.g., **inflammatory bowel disease**, **post–bariatric surgery**, **cystic fibrosis**, and other chronic malabsorptive disorders) **Chronic autoimmune diseases** (e.g., **rheumatoid arthritis**, **systemic lupus erythematosus, and multiple sclerosis**) **Skeletal disorders suggestive of deficiency**: osteoporosis, osteomalacia, or chronic skeletal/musculoskeletal pain **Chronic kidney disease** (especially advanced stages) and **chronic liver disease** **Hyperparathyroidism** **Granulomatous diseases** (e.g., **sarcoidosis**, **tuberculosis**, **histoplasmosis**, and other granuloma-forming disorders)

Overall agreement: 100% (consensus endorsed). Supplementary Table S1 provides the voting scale, level of agreement, and number of votes.

**Table 4:** Screening and Prevention of Vitamin D Deficiency in Adults.

We do not recommend routine community (population-wide) screening for vitamin D deficiency. Serum 25-hydroxyvitamin D [25(OH)D] should be measured in individuals at increased risk of vitamin D deficiency. In severe deficiency: In adults with 25(OH)D <12 ng/mL, measurement of serum total calcium (Ca), phosphate (P), parathyroid hormone (PTH), and alkaline phosphatase (ALP) is recommended to assess for secondary hyperparathyroidism and osteomalacia. In adults with 25(OH)D of 12–20 ng/mL and clinical features potentially attributable to vitamin D deficiency, measurement of Ca, P, iPTH, and ALP is recommended to evaluate for secondary hyperparathyroidism and osteomalacia. Symptoms suggestive of vitamin D deficiency include unexplained musculoskeletal or bone pain, proximal muscle weakness, muscle cramps, gait or balance impairment, and low-trauma (fragility) fractures. Prevention—general adult intake: The recommended dietary allowance (RDA) for vitamin D to support bone and muscle health in adults is 600 IU/day. Where dietary intake is inadequate (e.g., limited availability of vitamin D–fortified foods), supplementation of 600 IU/day may be considered. Prevention—older adults: Because cutaneous synthesis and intestinal absorption decline with age, 800 IU/day of vitamin D supplementation is recommended in older adults to support prevention of deficiency.

Overall agreement: 100% (consensus endorsed). Supplementary Table S1 provides the voting scale, level of agreement, and number of votes.

**Table 5:** Consensus Recommendations for Empiric Vitamin D Supplementation in High-Risk Populations.

**Adults at increased risk of vitamin D deficiency:**	
	Empiric vitamin D supplementation of 2000 IU/day is recommended, in addition to the estimated dietary requirement (approximately 800 IU/day, where achievable).
**Old population aged ≥75 years:**	
	Empiric vitamin D supplementation is recommended in adults aged ≥75 years, given evidence suggesting potential benefits on clinically relevant outcomes (including mortality). In this age group, in settings where vitamin D-fortified foods are limited, an additional 2000 IU/day of Vitamin D is recommended in addition to the dietary intake target (approximately 800 IU/day). In addition **to** the estimated dietary requirement (about 800 IU/day, where achievable), empiric supplementation with 2000 IU/day should primarily be considered for selected high-risk groups, particularly in settings where routine screening or food fortification is limited.
**Pregnancy**	
	Routine vitamin D screening is not recommended in pregnant women. Targeted/selective vitamin D screening may be considered for pregnant women with specific risk factors. Measurement of serum 25(OH)D should be considered only in pregnant women with ≥1 established risk factor for vitamin D deficiency. Given the potential to reduce the risk of adverse pregnancy outcomes (including preeclampsia, stillbirth, preterm birth, small-for-gestational-age birth, and neonatal mortality), empiric vitamin D supplementation is recommended during pregnancy. During pregnancy, empiric vitamin D supplementation of 2000–2500 IU/day is recommended in addition to dietary intake. Because most available clinical studies have evaluated daily dosing, a daily regimen is preferred. During lactation, a vitamin D intake of 2000 IU/day should be ensured.
**Prediabetes**	
	It is not advised for those with prediabetes to get routine vitamin D testing. Vitamin D supplementation may be explored as a supplement to lifestyle intervention in people with prediabetes, especially those who are at high risk of developing type 2 diabetes. Mean daily doses in clinical Trials have generally been around 3500 IU/day; any supplementation plan should be tailored based on baseline risk and stay under defined safe upper intake limits.

Overall agreement: 100% (consensus endorsed). Supplementary Table S1 provides the voting scale, level of agreement, and number of votes.

**Table 6:** Consensus Statements on the Treatment, Rapid Correction, Administration, and Monitoring of Vitamin D Deficiency in Adults.

	Consensus Statements
Treatment and targets	A serum 25-hydroxyvitamin D [25(OH)D] concentration <20 ng/mL should be considered an indication for treatment. Cholecalciferol (vitamin D3) is recommended for supplementation and treatment. For adults, a typical daily requirement is 800–2000 IU/day. When rapid correction (loading) is not required, a maintenance-oriented regimen of 800–2000 IU/day may be used. If underlying causes of vitamin D deficiency cannot be corrected, long-term maintenance supplementation may be required. In obesity, in patients receiving medications that accelerate vitamin D metabolism (e.g., systemic glucocorticoids and antiepileptic drugs), and in malabsorption syndromes, higher maintenance doses (approximately 3000–6000 IU/day) may be required. In persistent malabsorption, substantially higher doses (e.g., 10,000–50,000 IU/day) may be required; if response remains inadequate, hydroxylated forms of vitamin D may be considered. The tolerable upper intake level for routine daily supplementation in adults is 4000 IU/day. The treatment target is to maintain serum 25(OH)D between 20 and 50 ng/mL.
Rapid correction (loading)	A loading regimen is not routinely recommended but may be considered when rapid clinical correction is needed. Loading may be considered in adults with 25(OH)D <20 ng/mL and evidence suggestive of secondary hyperparathyroidism. Situations where rapid correction may be considered include very low 25(OH)D (<12 ng/mL), very high fracture risk in osteoporosis, secondary hyperparathyroidism, and hypocalcemia. Example regimen: 50,000 IU/week for 6–8 consecutive weeks, followed by 800–2000 IU/day maintenance. Alternative regimen: 6000–10,000 IU/day orally for 4 weeks, followed by 800–2000 IU/day maintenance. In obesity or malabsorption, a higher loading regimen (e.g., 100,000 IU/week for 8 weeks) may be considered, followed by maintenance (e.g., 4000–6000 IU/day) based on response.
Modes of administration	Daily and intermittent cumulative regimens (weekly, every two weeks, or monthly) have comparable efficacy and safety when equivalent cumulative doses are used. Capsule, drop, and tablet formulations demonstrate similar efficacy when used at equivalent doses. Parenteral vitamin D should be reserved for selected patients (e.g., severe malabsorption, inability to take oral therapy, or critically ill patients when clinically indicated). A patient-centered regimen incorporating dosing preferences (daily/weekly/monthly) may improve adherence.
Follow-up and monitoring	Serum 25(OH)D should be re-measured 8–12 weeks after initiation of treatment. Based on follow-up 25(OH)D concentrations, the regimen should be continued, adjusted, or transitioned to maintenance dosing. If 25(OH)D is within the target range (20–50 ng/mL), the same dose may be continued as maintenance therapy. If serum 25(OH)D is 50–60 ng/mL, concentrations are generally safe but exceed recommended targets; the dose and other sources of vitamin D should be reviewed. If serum 25(OH)D is 60–99 ng/mL (gray zone), the risk of complications may increase with higher concentrations; the regimen should be reviewed. Vitamin D can be stopped for 2–3 months; reconsider after measurement of 25(OH)D concentrations. If serum 25(OH)D is ≥100 ng/mL, it is defined as hypervitaminosis D; vitamin D intake should be stopped for at least 3 months, and clinical and biochemical evaluation should be undertaken. If serum 25(OH)D is ≥150 ng/mL, vitamin D intoxication (toxicity) should be considered. Vitamin D should be stopped for at least 3 months. Serum calcium levels should be measured and closely followed up.

Overall agreement: 100% (consensus endorsed). Supplementary Table S1 provides the voting scale, level of agreement, and number of votes.

## Data Availability

The data that support the findings of this study are available from the corresponding author upon reasonable request. We did not use artificial intelligence (AI) or AI-assisted technologies in the article.

## Supplementary Materials

The following supporting information can be downloaded at: https://www.mdpi.com/article/10.3390/nu18111665/s1, Table S1: Consensus voting scales according to consensus statements.

## Abbreviations

25(OH)D	25-hydroxyvitamin D
UVB	Ultraviolet B
UV index	Ultraviolet index
MED	Minimal erythemal dose
VDR	Vitamin D receptor
LC–MS/MS	Liquid chromatography–tandem mass spectrometry
CLIA	Chemiluminescence immunoassay
VDSP	Vitamin D Standardization Program
IOF	International Osteoporosis Foundation
iPTH/PTH	Intact parathyroid hormone/parathyroid hormone
ALP	Alkaline phosphatase
RDA	Recommended dietary allowance
DRI	Dietary reference intake
BMI	Body mass index
IOM	Institute of Medicine
IFG	Impaired fasting glucose
IGT	Impaired glucose tolerance
NGT	Normal glucose tolerance
T2DM	Type 2 diabetes mellitus
DPP	Diabetes Prevention Program
DPS	Finnish Diabetes Prevention Study
TEMD	Turkish Society of Endocrinology and Metabolism
