# Supplementary material for: Best Practice Recommendations for the Assessment, Prevention and Treatment of Vitamin D Deficiency in Türkiye: A 2026 Update in a Setting with Limited Mandatory Food Fortification
Source: Nutrients. 2026 May 22;18(11):1665. doi: 10.3390/nu18111665 (PMC13257591; doi:10.3390/nu18111665)
Supplement: Supplementary file 1 [file nutrients-18-01665-s001.zip › nutrients-4301669-supplementary.pdf]

Supplementary Table S1

Consensus statement	Level of Agreement	Consensus Voting Scale - percentage of votes
Vitamin D synthesis		
7-Dehydrocholesterol undergoes thermal photoisomerization under 290-315 nm UVB radiation (18 mJ/cm2) at temperatures ≥25 °C.	%90	9 (70%)<br>8 (10%)<br>7 (10%)<br>2 (10%)
In Turkey's geographical region (36°-42° north latitude), vitamin D synthesis takes place from early April to late September annually.	%90	9 (%40)<br>8 (20%)<br>7 (20%)<br>3 (10%)
Approximately 0.5 minimal erythemal dose of ultraviolet B radiation can be attained with direct sunlight exposure to the arms and legs; however, this is dependent upon factors such as time of day, season, latitude, skin type, advanced age, air pollution, and individual skin sensitivity.	%100	9 (90%)<br>8 (10%)
At the latitude of Türkiye, during the active UVB period between April and September, exposing the skin without sunscreen between 10:00 and 16:00 for 12 to 34 minutes, 3–4 times per week (depending on skin type), is sufficient to meet physiological vitamin D requirements	%100	9 (80%)<br>8 (10%)<br>7 (10%)
Sun exposure without sunscreen for longer than 15–30 minutes, depending on skin type, increases the risk of skin malignancies.	%100	9 (80%)<br>7 (10%)
Vitamin D screening		
The serum concentration of 25-hydroxyvitamin D [25(OH)D] is the accepted biomarker for evaluating vitamin D status.	%100	9-9 (90%)<br>5-1(10%)
The ideal method for measuring vitamin D is mass spectrometry. In settings where MS is not available, standardized immunoassays (antibody-based chemiluminescent or immunoenzymatic) should be used. Kits compliant with IOF standardization guidelines are recommended.	%100	9 (80%)<br>7 (20%)
A 25(OH)D level below 20 ng/mL should be considered vitamin D deficiency.<br>*A level below 12 ng/mL is classified as severe deficiency*	%100	9 (80%)<br>8 (10%)<br>7 (10%)
A 25(OH)D level of ≥20 ng/mL is considered sufficient for maintaining musculoskeletal health	%90	9(60%)<br>7(20%)<br>5(10%)<br>3(10%)
A 25(OH)D level between 30–50 ng/mL is considered sufficient for extra-skeletal effects	%100	9 (100%)
A 25(OH)D level between 30–50 ng/mL is considered sufficient for potential extra-skeletal effects.<br>*Levels between 50–60 ng/mL are safe but exceed the recommended targets.*<br>*Values between 60–99 ng/mL fall into a gray zone, where the risk of complications may increase with higher concentrations.*	%90	9 (90%)<br>6 (10%)
A 25(OH)D level above 100 ng/mL indicates vitamin D intoxication and carries a high risk of toxicity.	%100	9 (100%)
A 25(OH)D level above 150 ng/mL is defined as vitamin D intoxication.	%100	9 (100%)

Individuals at increased risk of vitamin D deficiency		
In our country's conditions, the population over 65 is at risk for vitamin D deficiency.	%90	9 (70%)<br>8 (10%)<br>7 (10%)<br>5 (10%)
People dependent on the home (disabled, those in nursing homes, immobile individuals)	%100	9 (100%)
Those who work indoors for long hours (such as those working night shifts in offices, hospitals, or factories)	%100	9 (100%)
Type 2 diabetes is at risk for vitamin D deficiency.	%100	9 (70%)<br>7 (30%)
People with chronic debilitating illnesses are at risk for vitamin D deficiency.	%90	9 (80%)<br>7 (10%)<br>5 (10%)
It is observed that the frequency of vitamin D deficiency in the adult population in Turkey is around 60%. This rate is higher than that of European and North American populations (12-30%).	%90	9 (50%)<br>8 (20%)<br>7 (10%)<br>5 (10%)
We do not recommend community screening for the diagnosis of vitamin D deficiency.	%100	9 (100%)
Vitamin D levels should be measured in individuals at risk of vitamin D deficiency.	%100	9 (100%)
In cases where vitamin D is below 12 ng/ml, it is recommended to check Ca, PTH, P, and ALP to rule out secondary hyperparathyroidism/osteomalacia.	%100	9 (100%)
In cases where vitamin D is below 20 ng/ml and there are symptoms associated with vitamin D deficiency, it is recommended to check Ca, PTH, P, and ALP to rule out secondary hyperparathyroidism/osteomalacia.	%90	9 (90%)<br>5 (10%)
Symptoms of vitamin D deficiency include unexplained muscle and bone pain, proximal muscle weakness, muscle cramps, imbalance, and fragility fractures.	%90	9 (50%)<br>8 (10%)<br>7 (30%)<br>5 (10%)
Prevention of Vitamin D Deficiency		
The recommended dietary allowance (RDA) for vitamin D for bone and muscle health in adults over 18 years of age is 600 IU, which is the minimum daily requirement.	%100	9 (70%)<br>7 (30%)
Although the daily vitamin D intake in our country is unknown, it is recommended to take 600 IU as a supplement because food is not fortified with vitamin D.	%100	9 (70%)<br>7 (30%)
Since the amount of vitamin D synthesis and absorption decreases in the elderly, a higher amount of RDA is required. 800 IU	%100	9 (70%)<br>7 (30%)
To prevent vitamin D deficiency in the elderly, and since it is not obtained thru food in our country, it is recommended to take 800 IU of vitamin D as a supplement, which is the recommended amount to be obtained thru food.	%100	9 (70%)<br>7 (30%)
**Empiric Vitamin D Supplementation**		
For those at risk of vitamin D deficiency, supplementation with 800-2000 IU of vitamin D is recommended to maintain 25OHD at target levels.	%100	9 (90%)<br>8-(10%)
For those at risk of vitamin D deficiency, supplementation with 2000 IU/day is recommended, along with the amount recommended to be obtained from food (800 IU).	%100	9-7 (70%)<br>7-3 (30%)
We recommend empirical vitamin D supplementation in the general population aged 75 and over due to its potential to reduce the risk of death.	%90	9 (60%)<br>8 (10%)<br>7 (20%)<br>6 (10%)

In the general population aged 75 and over, since there are not enough vitamin D-fortified foods available in our country, it is recommended to use an additional 2000 IU of vitamin D empirically, in addition to the 800 IU of vitamin D that should be obtained thru food.	%80	9 (30%)<br>8 (10%)<br>7 (40%)<br>5 (10%)<br>3 (10%)
**Pregnancy and Lactation**		
We do not recommend routine vitamin D screening for pregnant women.	%100	9 (100%)
Vitamin D measurement in pregnant women can only be performed if they have at least one of the risk factors for vitamin D deficiency.	%100	9 (100%)
Given the potential to reduce the risk of preeclampsia, intrauterine death, preterm birth, small-for-gestational-age birth, and neonatal death during pregnancy, we recommend empirical vitamin D supplementation.	%100	9 (100%)
In pregnant women, empirical vitamin D supplementation is recommended at 2000-2500 IU in addition to dietary intake.	%90	9 (50%)<br>8 (10%)<br>7 (30%)<br>4 (10%)
Since most current studies are conducted with daily doses, it is preferable for pregnant women to take their doses daily.	%100	9 (100%)
During lactation, a vitamin D intake of 2000 IU should be ensured.	%100	9(100%)
**Preventing vitamin D deficiency in other risk groups**		
Routine vitamin D screening is not recommended for prediabetic individuals.	%80	9 (80%)<br>5 (10%)<br>4 (10%)
In prediabetic individuals, vitamin D supplementation is recommended in addition to lifestyle changes to prevent the development of type 2 diabetes.	%80	9 (50%)<br>7 (30%)<br>6 (10%)<br>4 (10%)
Studies in prediabetic patients have shown that an average of 3500 IU of vitamin D is used. Therefore, an average of 3500 IU/day of vitamin D is recommended for high risk prediabetic patients to prevent disease progression.	%80	9 (50%)<br>7 (30%)<br>6 (10%)<br>4 (10%)
**Other chronic diseases**		
In chronic kidney disease, vitamin D supplementation is recommended for those with an estimated glomerular filtration rate (eGFR) >45 ml/min, just as it is for individuals with normal kidney function.	%100	9 (100%)
In chronic kidney failure, calcitriol should be used in addition to cholecalciferol in those with an estimated glomerular filtration rate (eGFR) < 45 ml/min.	%100	9 (100%)
To maintain 25OHD at target levels, vitamin D supplementation at 1000-2000 IU/day is recommended in chronic liver disease.	%100	9 (100%)
In recent cases of chronic liver disease, calcifediol can be added in situations like hypocalcemia secondary to hyperparathyroidism	%100	9 (100%)
**Treatment and Follow-up of Vitamin D Deficiency**		
A vitamin D level of <20 ng/ml requires treatment.	%100	9 (100%)
The goal of treatment is to keep the serum 25(OH)D vitamin level between 20-50 ng/ml.	%100	9 (100%)
The use of Cholecalciferol (D3) is recommended for supplementation and treatment	%100	9 (100%)

The daily optimal vitamin D requirement for adults is 800-2000 IU/day.	%90	9 (60%)<br>8 (30%)<br>5 (10%)
For those who will not receive a vitamin D load, treatment should be initiated at a dose of 1500-2000 IU/day.	%100	9 (100%)
As long as the causes of vitamin D deficiency cannot be eliminated, treatment must continue throughout life.	%100	9 (100%)
In obese individuals, those taking medications that accelerate vitamin D metabolism (corticosteroids, antiepileptic drugs), and in cases of malabsorption syndromes, the dose should be 2-3 times higher (3000-6000 IU/day maintenance).	%90	9 (80%)<br>7 (10%)<br>6 (10%)
Patients with malabsorption syndrome may require higher doses, such as 10,000 – 50,000 IU/day. If vitamin D deficiency/insufficiency persists despite these doses, better-absorbed hydroxylated forms of vitamin D can be tried. (calcitriol)	%100	9-5<br>7-5
The daily safe limit for vitamin D is 4000 IU/day.	%100	9 (100%)
**Rapid correction of vitamin D levels**		
A loading dose of vitamin D is not routinely recommended. Loading is recommended if a rapid clinical correction of vitamin D deficiency is needed. Vitamin D loading should be done for adults with 25(OH)D levels below 20 ng/ml if they have signs of secondary HPT.	%100	9 (100%)
situations where a rapid clinical correction of vitamin D is recommended include very low 25(OH)D levels <12 ng/dl, osteoporosis patients with a very high risk of fracture, secondary hyperparathyroidism, and hypocalcemia.	%100	9 (100%)
Adults with 25(OH)D levels below 20 ng/ml should undergo vitamin D loading if they exhibit secondary HPT findings.	%100	9 (100%)
Vitamin D loading should be followed by a maintenance dose of 1500-2000 IU per day after 50,000 IU of vitamin D is administered once a week for 6-8 consecutive weeks.	%90	9-8,7-1<br>6-1
Alternatively, a regimen can be recommended with a loading dose of 6000-10000 IU per day, administered orally for 4 weeks, followed by a maintenance dose of 800-2000 IU per day	%90	9-8,7-1<br>6-1
In cases of obesity and malabsorption, the loading dose is 100,000 IU/week, administered for 8 weeks, after which it can be continued with 4000-600 IU/day.	%90	9-8,7-1<br>6-1
serum 25(OH)D levels should be measured 8-12 weeks after treatment is initiated. If the target 25(OH) vitamin D level has not been reached, an additional dose can be given.	%80	9-3,8-2,7-3,<br>5-1 ,3-1
Follow-up and Monitoring of Patients Undergoing Treatment for Vitamin D Deficiency		
In patients who have started treatment for vitamin D deficiency, serum 25(OH)D levels should be measured 8–12 weeks after initiation of therapy.	%80	9-3,8-2<br>7-3,5-2
Based on the result, a decision should be made either to continue the current treatment regimen or to switch to a maintenance dose.	%100	9-10
The treatment target is to maintain serum 25(OH)D levels between 20–50 ng/mL. If the measured level is within this target range, the same dose should be continued as maintenance therapy.	%90	9-7,8-2,6-1
If serum 25(OH)D concentrations are between >50 and 100 ng/mL, the appropriateness of the previous treatment regimen should be verified; intake, dosage, and adherence should be assessed and the regimen revised accordingly.	%90	9-5,7-4<br>6-1
If the serum 25(OH)D level is >50–75 ng/mL, the dose should be reduced, and the vitamin D level should be re-measured after 2 months	%100	9-5,8-1<br>7-4

ıf the serum 25(OH)D level is 75–100 ng/mL, vitamin D therapy should be withheld for 2–3 months; treatment should be reinitiated with an adjusted dose once the 25(OH)D level falls below 50 ng/mL.	%100	9-5<br>8-1<br>7-4
Modes of Vitamin D Administration		
Daily and cumulative dosing regimens (weekly, every 15 days, or monthly) have comparable efficacy and safety.	%70	9-4,7-3<br>5-1,3-2
Capsule, drop, and tablet formulations demonstrate similar efficacy when used at equivalent doses	%100	9-10
Parenteral administration is recommended only in patients with gastrointestinal malabsorption who are resistant to oral therapy, in those who cannot take oral medication, and in critically ill intensive care patients	%100	9-10
An individualized treatment strategy that takes into account patients' preferences for dosing regimens (daily, weekly, or monthly) may improve adherence to vitamin D therapy	%90	9-8<br>7-1<br>3-1
**Use of Vitamin D Analogs**		
Calcitriol and other active vitamin D analogues should not be used to prevent vitamin D deficiency. They should be reserved for specific treatment indications and used in conditions with impaired intrinsic vitamin D metabolism, such as chronic kidney disease or hypoparathyroidism.	%100	9-10
Serum 25(OH)D is not an appropriate marker for monitoring therapy with active vitamin D analogues. If needed, $1,25(OH)_2D$ levels may be used.	%100	
Treatment with active vitamin D analogues requires monitoring of serum calcium, phosphate, and alkaline phosphatase, as well as 24-hour urinary calcium excretion.	%100	9-6,8-1<br>7-3
Calcifediol may be used instead of cholecalciferol in conditions such as chronic liver disease or malabsorption.	%100	
**vitamin D in Musculoskeletal Diseases**		
In patients with osteoporosis, it is recommended to administer oral cholecalciferol supplementation at a dosage of 800–2000 IU/day, in conjunction with calcium.	%100	9-7<br>7-3
It is advised to screen serum 25(OH)D levels and treat vitamin D deficit prior to starting antiresorptive treatment for osteoporosis since vitamin D deficiency may affect the response to osteoporosis therapy.	%100	9-10
Falls or fracture-risk–increased patients are recommended to receive 800–2,000 IU/day of supplementation	%90	9-5<br>8-1<br>7-3<br>2-1
During the prevention and treatment of vitamin D deficiency, adequate dietary calcium intake should be ensured, taking into account sufficient hydration/rehydration.	%100	9-7<br>8-1<br>7-2
if adequate calcium intake cannot be achieved through diet, calcium salt supplements are recommended, preferably in divided doses, and should be taken with meals while ensuring appropriate hydration.	%100	9-7<br>8-1<br>7-3

Consensus voting scale : 9 (strongly agree),8,7 (agree),6,5(neutral),4, 3(disagree),2, 1(strogly disagree). Percentace of number of votes presented with scale numbers
